# Metagenomic profiling of viral and microbial communities from the pox lesions of lumpy skin disease virus and sheeppox virus-infected hosts

**DOI:** 10.3389/fvets.2024.1321202

**Published:** 2024-02-14

**Authors:** Fedor S. Sharko, Ali Mazloum, Alena O. Krotova, Olga P. Byadovskaya, Larisa B. Prokhvatilova, Ilya A. Chvala, Ustin E. Zolotikov, Alexandra D. Kozlova, Anastasia S. Krylova, Erika V. Grosfeld, Anastasia V. Prokopenko, Aleksei A. Korzhenkov, Maxim V. Patrushev, Zorigto B. Namsaraev, Alexander V. Sprygin, Stepan V. Toshchakov

**Affiliations:** ^1^National Research Center “Kurchatov Institute”, Moscow, Russia; ^2^Federal Center for Animal Health FGBI ARRIAH, Vladimir, Russia; ^3^Moscow Institute of Physics and Technology, National Research University, Dolgoprudny, Russia

**Keywords:** lumpy skin disease virus, sheep pox virus, metagenome, bacterial community, shotgun sequencing, skin microbiome, skin lesion microbiome

## Abstract

**Introduction:**

It has been recognized that capripoxvirus infections have a strong cutaneous tropism with the manifestation of skin lesions in the form of nodules and scabs in the respective hosts, followed by necrosis and sloughing off. Considering that the skin microbiota is a complex community of commensal bacteria, fungi and viruses that are influenced by infections leading to pathological states, there is no evidence on how the skin microbiome is affected during capripoxvirus pathogenesis.

**Methods:**

In this study, shotgun metagenomic sequencing was used to investigate the microbiome in pox lesions from hosts infected with lumpy skin disease virus and sheep pox virus.

**Results:**

The analysis revealed a high degree of variability in bacterial community structures across affected skin samples, indicating the importance of specific commensal microorganisms colonizing individual hosts. The most common and abundant bacteria found in scab samples were *Fusobacterium necrophorum*, *Streptococcus dysgalactiae*, *Helcococcus ovis* and *Trueperella pyogenes*, irrespective of host. Bacterial reads belonging to the genera *Moraxella*, *Mannheimia*, *Corynebacterium*, *Staphylococcus* and *Micrococcus* were identified.

**Discussion:**

This study is the first to investigate capripox virus-associated changes in the skin microbiome using whole-genome metagenomic profiling. The findings will provide a basis for further investigation into capripoxvirus pathogenesis. In addition, this study highlights the challenge of selecting an optimal bioinformatics approach for the analysis of metagenomic data in clinical and veterinary practice. For example, direct classification of reads using a kmer-based algorithm resulted in a significant number of systematic false positives, which may be attributed to the peculiarities of the algorithm and database selection. On the contrary, the process of *de novo* assembly requires a large number of target reads from the symbiotic microbial community. In this work, the obtained sequencing data were processed by three different approaches, including direct classification of reads based on k-mers, mapping of reads to a marker gene database, and *de novo* assembly and binning of metagenomic contigs. The advantages and disadvantages of these techniques and their practicality in veterinary settings are discussed in relation to the results obtained.

## 1 Introduction

The capripoxviruses are highly contagious and often fatal viral infections of sheep and cattle. They are notifiable to the World Organization of Animal Health (WOAH) (1). These viruses belong to the *Poxviridae* genus *Capripoхvirus*, which includes sheep pox virus (SPV), goat pox virus (GTPV) and lumpy skin disease virus (LSDV) (2, 3). Being firstly discovered in Africa, currently *Capripoxvirus-*related infections were first discovered in Africa and have since rapidly spread to Europe and Asia (4). The genomes of *Capripoxviruses* consist of double-stranded DNA of approximately 150 kilobase pairs in length, with terminal repeats at each end, encoding 147 open reading frames (ORFs) (3). The genomes of all three species are closely related, showing 97% nucleotide identity. The high level of similarity between poxviruses increases the likelihood of recombination in the field (5, 6). However, capripoxviruses exhibit varying levels of host adaptation. While LSD occurrences have been observed in antelopes and giraffes, their primary hosts are cattle and water buffaloes (7). In turn, GTPV and SPPV have been found to possess natural or experimental cross-infection capability, causing sickness in both host species, goats and sheep (8).

Affected sheep and goats develop fever and varying degrees of generalization. Eyelids become swollen and mucopurulent discharge crusts the nostrils. Widespread skin lesions develop that are readily seen on the muzzle, ears, and wool free areas (9). Lesions start as erythematous areas on the wool free skin and progress rapidly to raised, circular plaques with congested borders (9). Upon necropsy, observations often showed lung oedema and congestion, as well as nodules present throughout the lungs and under the skin into the muscles (10). As lesions start to regress, necrosis occurs, followed by the formation of scabs that are abundant in viral particles (11). Under experimental conditions, up to 50% of capripoxvirus-infected animals do not develop the typical nodules and remain uninfected or subclinically infected (12).

Despite the increasing amount of research on capripoxviruses, there are still considerable gaps in knowledge, especially concerning genetic diversity, host specificity, transmission pathways, and the differences in symptom severity between field outbreaks and experimental settings (12–15). The skin typically harbors different bacterial and viral communities that can serve as subjects for metagenomic research under both natural and experimental conditions (16). However, despite skin lesions or scabs being the characteristic feature of capripoxvirus diseases, there is a lack of published knowledge regarding the bacterial and viral communities present in capripox-induced lesions. Investigating potential coinfections and identifying novel agents during capripoxvirus infection could yield valuable knowledge of the underlying natural pathogenesis mechanisms. It is important to consider the likelihood of secondary bacterial and viral infections, which commonly arise as complicating factors for capripoxviruses, thus requiring antibiotic treatment (17).

Metagenomic approaches are progressively utilized in clinical (18, 19) and veterinary practice for monitoring co-infections and opportunistic infections. Apart from direct co-infection monitoring, metagenomic methods have been successfully used to analyze host specificity and genetic diversity of both novel and characterized etiological agents. The review by Suminda and co-authors in 2022 provides further information on this topic (20). Another application of metagenomic approach is the analysis of transmission pathways of zoonotic infections, which can be performed even using environmental samples taken outside of the host (21). The most cost-effective and suitable method for monitoring of co-infections and generalized analysis of microbial community composition is the highly parallel sequencing of marker genes (e.g., 16S rRNA). However, this method has its limitations, including low taxonomic resolution and the ability to assess only a specific group of pathogens, leading to restricted interpretation of findings (22). Shotgun metagenomic sequencing enables simultaneous analysis of bacteria, viruses, and eukaryotic microorganisms in one dataset, providing high precision phylogenetic analysis (23). The main challenge with this technique is the choice of a reliable and data-efficient algorithm for microbial community analysis (23). On one hand, direct read classification necessitates minimal data and enables identification of sample community composition; however, on the other hand, this approach frequently yields false-positive results (24). On the other hand, *de novo* assembly of microbial genomes enables highly accurate determination of phylogeny and analysis of potential antibiotic resistance (25). However, this approach necessitates extensive data and multiple samples for differential coverage binning of metagenomic contigs.

As previously stated, the objective of this study is to analyze the microbiome present in skin scabs induced by SPV/LSDV using shotgun metagenomic sequencing. In this study three independent approaches were utilized to analyze metagenomic data obtained by sequencing of clinical samples, namely the classification of reads through k-mers, classification of reads via mapping to a marker gene database, and *de novo* assembly and binning of metagenomic contigs. The benefits and drawbacks of these bioinformatic methods for clinical applications are discussed based on the findings obtained.

## 2 Materials and methods

### 2.1 Samples description

Twelve skin scab samples were taken from animals showing symptoms of SPPV infection from different regions of the Russian Federation and from different hosts (*Bos taurus* and *Ovis aries*). Samples were shipped to the reference laboratory at the Federal Institute for Animal Health (FGBI “ARRIAH”) for the molecular confirmation of SPPV or LSDV infection. The selection criteria for the samples were as follows: they had to be delivered to FGBI ARRIAH on cold ice and in sufficient quantity (at least 200 mg of biomass). The cold chain is crucial for preserving the microbial community inherent to a living animal, without any artifacts caused by the overgrowth of secondary microflora. As samples are frequently obtained from remote regions with poor logistics, the cold chain is rarely respected. Therefore, we were only able to collect three sheep and nine cattle samples for this study. All 12 samples tested positive for SPPV genome using an in-house PCR kit (26) (Table 1).

**Table 1:** Sample characteristics.

Sample ID	Sample collection region	Year of sample collection	Host
LSDV_88	Chelyabinsk	2018	*Bos taurus*
LSDV_89	Kurgan	2018	*Bos taurus*
LSDV_90	Samara	2018	*Bos taurus*
SPV_92	Pskov	2019	*Ovis aries*
SPV_93	Moscow	2018	*Ovis aries*
SPV_94	Amur	2018	*Ovis aries*
SPV_95	Tver	2019	*Ovis aries*
SPV_96	Dagestan	2022	*Ovis aries*
SPV_97	Moscow	2018	*Ovis aries*
SPV_98	Kaluga	2020	*Ovis aries*
SPV_99	Tula	2018	*Ovis aries*
SPV_100	Kostroma	2021	*Ovis aries*

### 2.2 DNA extraction and fragment library preparation

Metagenomic DNA was extracted with QIAamp DNA Microbiome Kit (Qiagen, Germany), according to manufacturer’s instructions. The specified kit was chosen because it allows enrichment of the microbial fraction of the sample by differential lysis of eukaryotic cells. Quantity and quality of DNA was assessed using NanoDrop^™^ 8,000 Spectrophotometer (Thermo Fisher Scientific, Carlsbad, CA, United States). The A260/280 ratio was in the range of 1.8–1.9, while A260/230 was in the range of 1.8–2.2, reflecting sufficient purity of the DNA preparations. 400 nanograms of DNA from each sample was used as an input for library preparation. DNA was fragmented to 250–320 bp fragments with Covaris ME220 Focused-ultrasonicator (Covaris, Woburn, MA, United States) according to sonication protocol, provided by the manufacturer. The ultrasonic fragmentation method was selected because it minimizes biases in microbial community composition that may arise from suboptimal fragmentase performance for AT or GC-rich genomes. Standard fragment DNBSeq-compatible libraries were prepared using MGIEasy Universal DNA Library Prep kit and further circularized with MGIEasy Circularization Kit (both—MGITech, People’s Republic of China), according to the instructions provided by the manufacturer.

### 2.3 Sequencing and quality control

Sequencing was performed by MGITECH DNBSEQ 400 platform (MGITECH, People’s Republic of China), which showed excellent results in benchmark metagenomic studies (27). For the sequencing DNBSEQ-G400RS High-throughput Sequencing Set (PE150) reagent kit was used, since it was the maximum readlength available at the time of experiments. Demultiplexing of indexed samples was performed on the instrument. Quality control was performed by the analysis of raw read quality data with seqkit (28) and FastQC tools (29).

### 2.4 Community profiling by direct classification of metagenomic reads

Obtained metagenomic reads were trimmed and filtered by quality with fastp version 0.23.4 (30) using the following parameters: mimal average quality in front and tail 4 bp sliding window—25; maximum number of unidentified bases in the read—1; minimal average quality in trimmed read—20; minimal length of trimmed read—50. Host reads and reads, corresponding to human DNA contamination were detected by the mapping of reads to the host or contamination genome (*Homo sapiens*, *Bos taurus* and *Ovis aries*) using Bowtie2 (31).

Before using the Kraken2 classifier, a custom database was created, including RefSeq archaea, bacteria, viruses, protozoa, fungi plasmid complete genomes, UniVec Core and mammalian genomes, including human, cow and sheep genomes. This database was used for the Kraken2 to classify all reads, providing each read with its taxonomy identification. To obtain reliable classification results using the Kraken2, parameters of confidence = 0.3 and minimum-hit-groups = 3 were used. Bracken was used to estimate species abundance in each sample with parameters: -r 100 -t 50 (32). Using the KrakenTools alpha_diversity.py script, the Berger-Parker, Fisher, Simpson, inverse Simpson, and Shannon α-diversities were calculated for each sample (33).

For the classification of reads by clade-specific marker genes with Metaphlan4 (34) timed and filtered sequencing read pairs were classified against last version of Metaphlan4 database with standard classification parameters.

### 2.5 *De novo* assembly, metagenomic binning and phylogenetic analysis

Each sequenced sample was assembled separately. Trimmed and filtered reads were assembled with metaSPAdes *de novo* assembler (35) using k-mer sizes of 99 and 127. The choice of a higher kmer length compared to the default values implemented in SPAdes for metagenomic assembly was due to the fact that the read length was 150 nucleotides, allowing higher values to be used in order to obtain a better assembly (36). Binning of metagenomic contigs was performed by Concoct (37), Metabat2 (38) and Maxbin 2.0 (39) algorithms, implemented in Metawrap pipeline (40). Additional binning was performed with in-house developed pyYamb binning tool (41). Bin refinement of obtained bin sets was performed with DAS Tool package (42). Taxonomy of resulting bins was determined with GTDB-toolkit (43). Completeness and contamination level of resulting bins was estimated with CheckM2 package (44).

41 marker gene phylogenetic tree was reconstructed as follows: Markov models of marker genes were acquired from the Pfam database (45). Genes were searched by amino acid sequences utilizing hmmscan v.3.3.2 with an e-value of 1e-10 (46). The corresponding nucleotide sequences for each marker were automatically aligned using MAFFT v.7.520 (47). The phylogenetic tree was developed using the GTR evolutionary model with Fasttree v.2.1.11 (48) with 1,000 bootstraps.

Extraction of sequences used for MLST of *S.dysgalactiae* was performed with FastMLST package (49). Alignment and phylogeny reconstruction was performed with mafft (47) and IQTree with 1,000 botstraps (50), respectively. All phylogenetic trees were visualized with iTOL web server (51). ANI analysis and heatmap visualization was performed with pyANI package (52).

Analysis of antibiotic resistance genes in obtained metagenomic bins was performed with *staramr* package (53).

## 3 Results

### 3.1 Analysis of diversity of skin scabs by direct metagenomic classification of reads

#### 3.1.1 Metagenomic sequencing and host contamination

The study analyzed the microbiome of skin scabs from lumpy skin disease and sheep pox using samples submitted to FGBI ARRIAH from various regions of Russia for PCR confirmation of capripoxvirus infections. Strict selection criteria were applied to the condition of biomass specimens upon receipt due to the possibility of secondary microflora overgrowth in samples taken from non-sterile farm conditions (see Methods). Due to that fact, only three samples of cattle scab biomass and nine samples of sheep scab were selected (Table 1).

Sequencing of shotgun metagenomic libraries with DNBSeq-400 sequencer resulted in 10.2 to 34.3 million of read pairs per sample. Removal of host reads revealed high levels of contamination with host DNA ranging between 57.0 and 96.7% (Table 2). Additional filtering of human-related reads, recommended for farm and domestic animals (54), showed the contamination with human DNA at the 0.15–2.56% (0.64% on average). Following quality filtering and removal of host- and human-related contamination, 0.31–9.15 million read pairs were available for direct classification and *de novo* assembly.

**Table 2:** Processing of metagenomic reads.

Sample ID	Raw reads	Quality filtered reads	Reads mapped to host genome	Reads mapped to human DNA	Reads used for the analysis
LSDV_88	10.18	0.21 (2.06%)	9.15 (89.88%)	0.04 (0.39%)	0.78 (7.66%)
LSDV_89	32.91	0.37 (1.12%)	23.15 (70.34%)	0.14 (0.43%)	9.25 (28.11%)
LSDV_90	26.03	0.45 (1.73%)	23.86 (91.66%)	0.05 (0.19%)	1.67 (6.42%)
SPV_92	26.77	0.39 (1.46%)	12.78 (47.74%)	0.11 (0.41%)	13.49 (50.39%)
SPV_93	11.17	0.21 (1.88%)	5.32 (47.63%)	0.11 (0.98%)	5.53 (49.51%)
SPV_94	22.49	0.36 (1.6%)	10.26 (45.62%)	0.07 (0.31%)	11.8 (52.47%)
SPV_95	26.01	0.32 (1.23%)	11.83 (45.48%)	0.21 (0.81%)	13.65 (52.48%)
SPV_96	32.27	0.63 (1.95%)	11.58 (35.88%)	0.06 (0.19%)	20 (61.98%)
SPV_97	13.73	0.45 (3.28%)	6.77 (49.31%)	0.13 (0.95%)	6.38 (46.47%)
SPV_98	15.21	0.30 (1.97%)	4.49 (29.52%)	0.4 (2.63%)	10.02 (65.88%)
SPV_99	34.33	0.5 (1.46%)	17.19 (50.07%)	0.3 (0.87%)	16.34 (47.6%)
SPV_100	11.52	0.19 (1.65%)	4.15 (36.02%)	0.1 (0.87%)	7.08 (61.46%)

The number of reads is given in millions. Percentage of raw reads is given in brackets.

#### 3.1.2 Alpha-diversity

**Figure 1:**
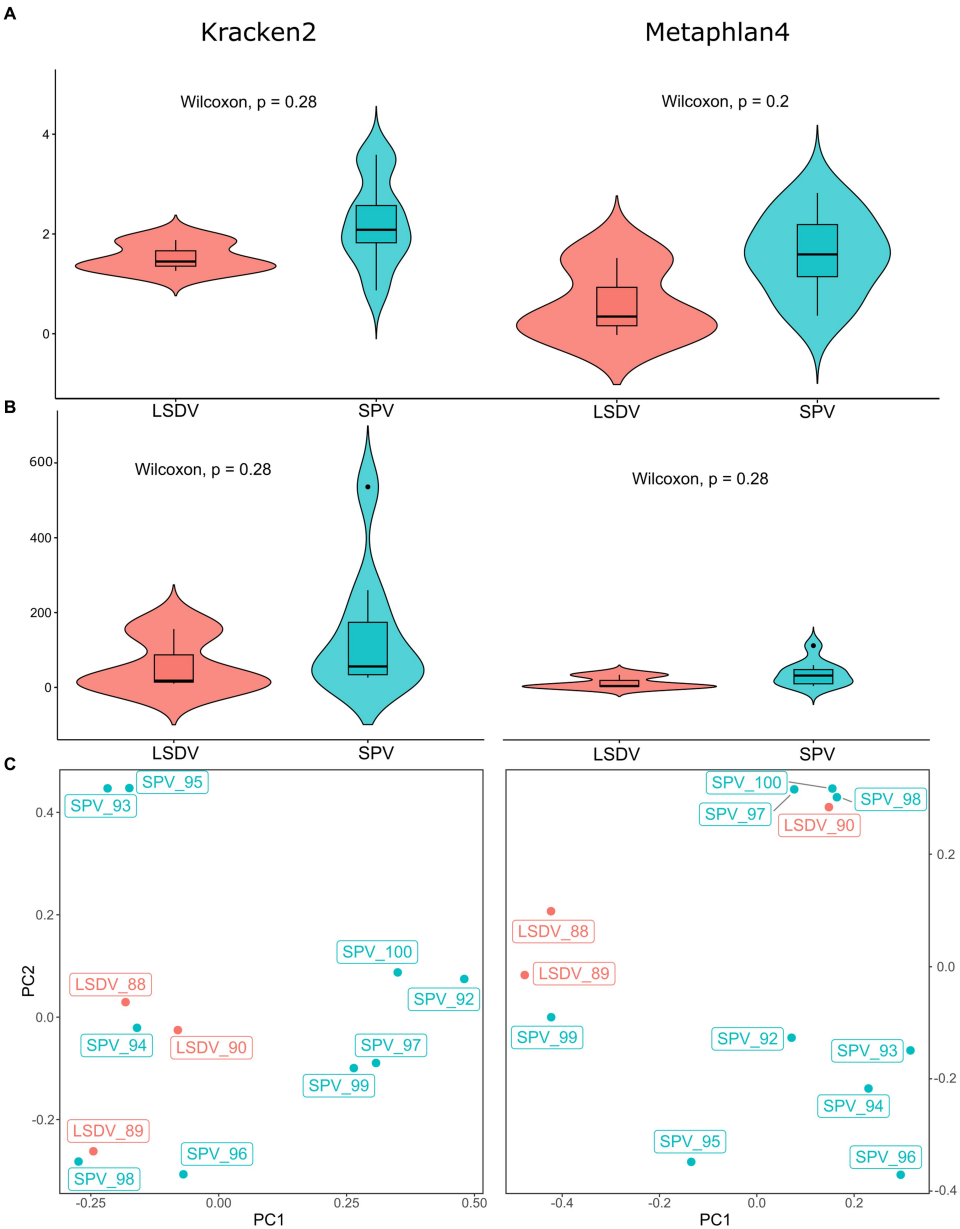
Alpha and beta diversity of microbial communities from skin scabs of LSDV- and SPV-infected animals. **(A)** Violin plots of Shannon indexes compared between LSDV- and SPV-infected animals. Indexes based on Kraken2 results are shown on the left panel, indexes based on Metaphlan4 are shown on the right panel. **(B)** Violin plots of number of species compared between LSDV- and SPV-infected animals. Values based on Kraken2 results are shown on the left panel, values based on Metaphlan4 are shown on the right panel. **(C)** Principal component analysis of microbial communities of skin scabs.

Direct metagenomic classification of reads, performed with Kraken2 (55) using customized RefSeq database supplemented with host genomes (see Methods), resulted in classification of 14.7–67.2% of filtered reads (Supplementary Table 1). However, despite pre-filtering of reads by mapping to the host genome, 5.35–28.71% of filtered reads were classified as host-related. It should be noted that 5.35–28.71% of filtered reads were classified as host-related. To verify Kraken2’s findings by different classification algorithm, the data was cross-checked using the Metaphlan4 package. This package implements an algorithm that relies on read mapping to single-copy marker genes (34). Marker genes represent a small fraction of the genome, resulting in a lower percentage of classified reads for Metaphlan4 compared to Kraken2. Consequently, Metaphlan4 detects underrepresented microorganisms poorly, leading to lower alpha diversity metrics than Kraken2. At the same time, alpha diversity metrics for samples with over 10 million target reads were comparable between Kraken2 and Metaphlan4 (Figure 1; Supplementary Table 2).Several benchmark studies have found that Metaphlan4 exhibits greater specificity but lower sensitivity than Kraken2 (56). Consequently, the alpha diversity metrics based on Metaphlan4 were significantly lower than those derived from Kraken2 results, with the average Shannon index value of 1.38 for Metaphlan4 and 2.06 for Kraken2. The average number of detected species was also much lower, with 16 for Metaphlan4 and 61 for Kraken2 (Figure 1). Alpha diversity metrics showed no significant differences between LSDV-infected animals and SPV-infected animals (Figure 1). Unfortunately, due to specifics of sample collection procedure, comparison of microbiota diversity level for healthy and affected skin areas for each animal was not possible. Several studies on the microbiome of skin lesions in humans indicate a decrease in alpha diversity in affected areas due to the prevalence of opportunistic microorganisms (57, 58). Although there is limited research on skin diseases in farm animals, there is evidence of decreased diversity in skin lesions caused by udder cleft dermatitis in dairy cows (59). This could partly account for the low alpha-diversity metrics observed in this study.

#### 3.1.3 Beta-diversity

PERMANOVA analysis of beta diversity using *adonis2* function of *vegan* package revealed no significant differences between LSDV- and SPV-infected animals, neither for Kraken2 (*R^2^* = 0.114, value of *p* > 0.1, with 999 permutations) nor for Metaphlan4 (*R^2^* = 0.126, value of *p* > 0.01, with 999 permutations). Principal component analysis of results also had not shown distinct sample clusterization (Figure 1; Supplementary Table 4). This result indicates that the structure of the microbial community present in scabs is mainly determined by chance, influenced by the prevalence of a specific bacterial opportunistic pathogen within the lesion area. However, it is not affected by the specific type of capripoxvirus present.

### 3.2 Microbiome composition of skin scabs

#### 3.2.1 Core microbiome

**Figure 2:**
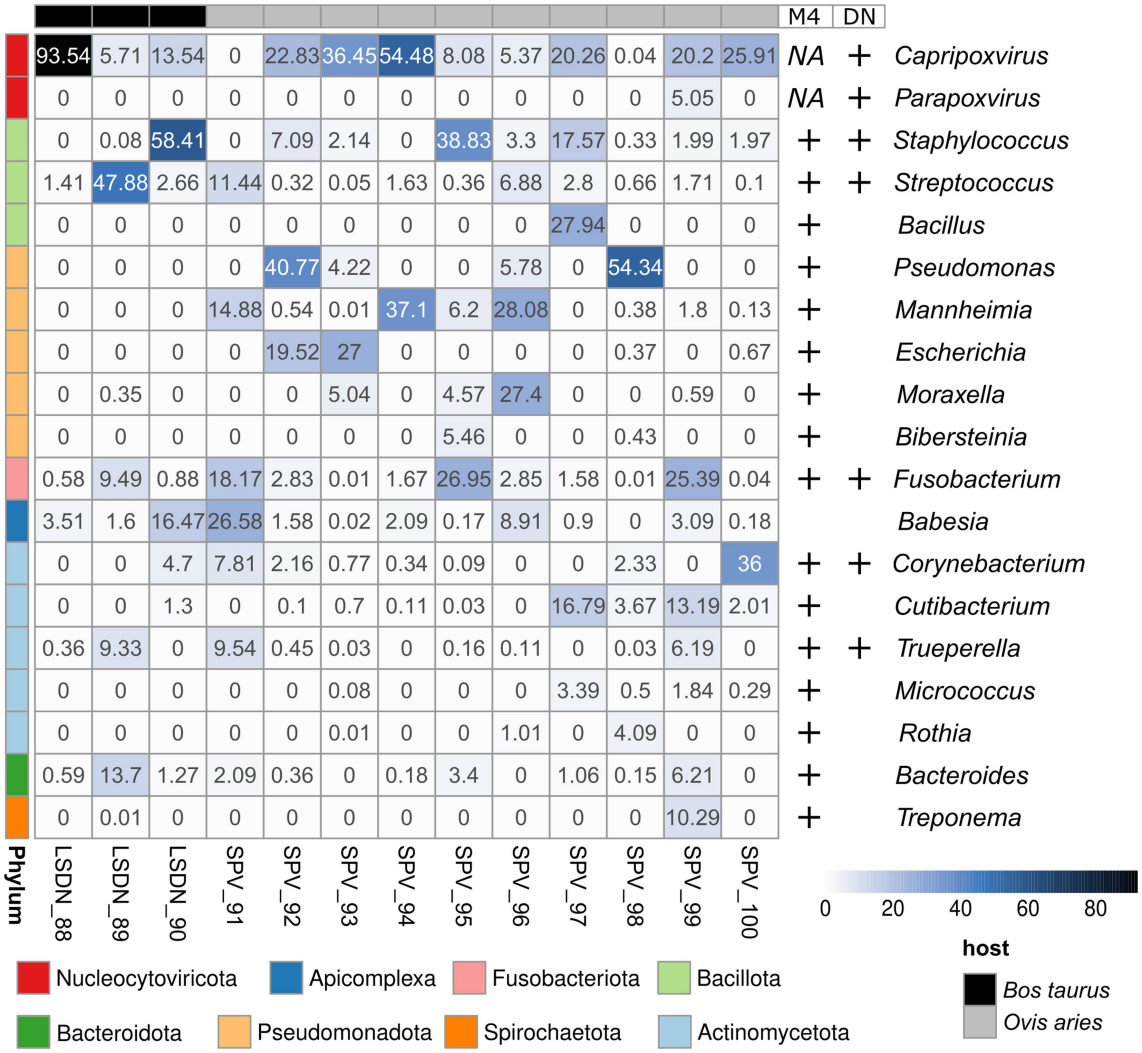
Abundance heatmap of Kraken2-classified reads. Abundance values shown in per cents. Results, supported by Metaphlan4 (M4) or *de novo* metagenomic assembly and bin classification (DN) are highlighted with ‘+’ signs at the right side of the heatmap.

Kraken2-classified sequencing reads were distinctly prevailed by those, corresponding to main infection agent (Figure 2). Notably, the classification of a small number of reads to SPV in LSDV-infected animals and vice versa was observed. However, *de novo* assembly analysis did not confirm the presence of the two viruses, which allows us to attribute this result to false positives caused by the low specificity of the kmer-based algorithm implemented in Kraken2. Interestingly, one case of viral co-infection of sheep (sample SPV_99) with orf virus (60) was discovered (Figure 2).

The only eukaryotic microorganism identified was *Babesia bigemina*, detectable in all samples analyzed. The number of reads assigned to it ranged from 0.003 to 16.5% of all Kraken2-classified reads (Figure 2; Supplementary Figure 1). However, the results from Metaphlan4 did not support this finding (Supplementary Figure 2; Table 3). Further analysis of the reads assigned to *Babesia bigemina* by Kraken2 showed that they were all mapped to the small scaffold of the *B.bigemina* genome, but not to the chromosomes (Supplementary Table 5). That fact might be explained by prokaryotic contamination of *B.bigemina* assembly, deposited in NCBI RefSeq and imported in Kraken2 database.

The dominant prokaryotic microorganisms varied from sample to sample, according to Kraken2/Metaphlan4 (Figure 2; Supplementary Table 3; Figures 1, 2). Analysis of the core members of the communities showed that the only species, detected by all tools in more than 50% of samples with an abundance greater than 1% was *Fusobacterium necrophorum,* reported to cause foot rot disease in cattle (61) and sheep (62) (Table 3). Other core taxa varied according to sample group (SPV/LSDV) and method of analysis (Kraken2/Metaphlan4). For LSDV-infected animals *Streptococcus dysgalactiae,* known to cause mastitis in cattle (63) was detected by Kraken2 in all samples, however only in samples of LSDV-infected animals that microorganisms prevailed (Figure 2). In turn, 7 out of 9 sheep samples demonstrated the presence of the common opportunistic pathogen *Staphylococcus aureus* capable of causing the development of mastitis and other skin diseases in livestock (64, 65). *Bacteroides heparinolyticus*, reported to be associated with metritis of dairy cows (66) was detected in 9 of 12 samples analyzed. SPV-infected sheeps showed significant increase of abundance of *Cutibacterium*, compared to cows (Figure 2). In turn, Metaphlan4 results showed the presence of *Helcococcus ovis* in 2 of 3 LSDV scab samples and 3 of 9 SPV samples. Interestingly, according to Metaphlan4, *H. ovis* absolutely dominated the microbial community in sample LSDV_88, reaching 100% of classified prokaryotic reads (Supplementary Figure 2). That bacteria, firstly isolated from lung and liver of *postmortem* sheep (67) was recently reported to be associated with sheep lung diseases (68) and to cause abortions in cattle (69). Detection of *H. ovis* was supported by genome-based taxonomic classification of *de novo* assembled metagenomic bins, therefore we suppose that result as false negative of Kraken2. Thus, all the bacteria identified as core community members of capripoxvirus-associated skin lesions, were previously reported to be associated with skin diseases of the livestock.

**Table 3:** Core members of microbiota of capripoxvirus-associated scars.

	LSDV-infected animals	SPV-infected animals
core members identified by Kraken2	*Fusobacterium necrophorum* *Streptococcus dysgalactiae* *Bacteroides heparinolyticus*	*Fusobacterium necrophorum* *Staphylococcus aureus* *Cutibacterium acnes* ^1^
core members identified by Metaphlan4	*Fusobacterium necrophorum* *Helcococcus_ovis*	*Fusobacterium necrophorum*

^1^Potential false positive.

#### 3.2.2 Sample-specific microbiome

**Figure 3:**
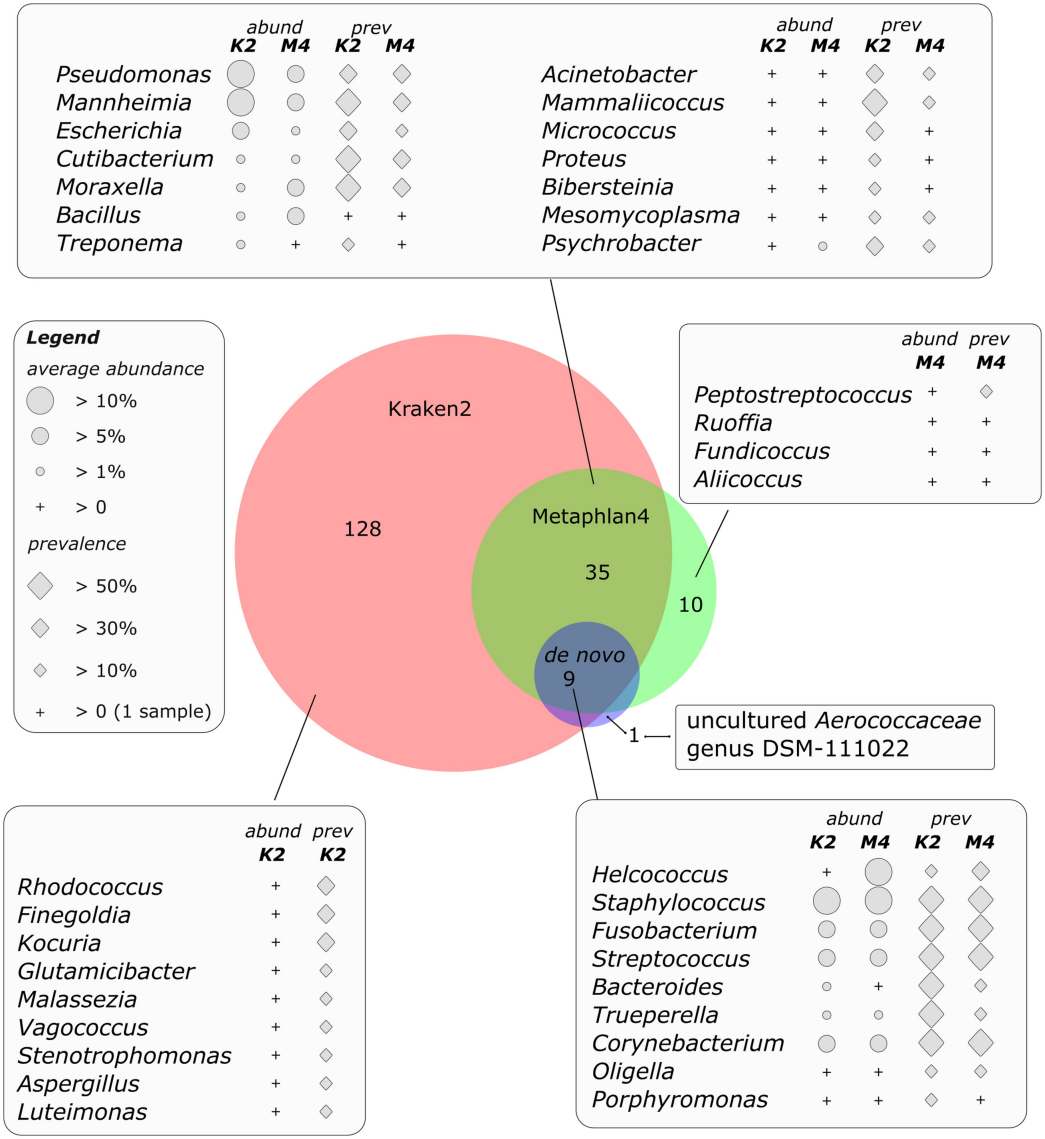
Area proportional Venn diagram of genera detected by different bioinformatic approaches. Diagram was drawn using DeepVenn web application (70).

Members of the scab microbial community sporadically detected in different samples were highly diverse. At the genus level, 172 genera were detected using Kraken 2. At the same time, a more specific marker gene analysis using Metaphlan4 allowed the detection of only 54 valid genera. *De novo* assembly and subsequent binning of metagenomic contigs allowed to obtain 10 metagenomic baskets of acceptable quality. Thus, 44 microbial genera were detected by at least two of the three approaches used (Figure 3).

The above-mentioned members of the core part of the microbial community namely *Fusobacterium*, *Streptococcus*, *Staphylococcus, Bacteroides* and *Helcococcus* were detected by all the three methods of analysis. The abundance and prevalence of these bacteria were comparable for all approaches, except of *Helcococcus*, whose abundance was supposedly undestimated by Kraken2 due to the absence of *Helcococcus ovis* genome in the reference database (some reads were classified by Kraken2 as *Helcococcus bovis*). Other bacteria, detected by both direct classification of sequencing reads and by *de novo* assembly and binning belonged to the genera *Trueperella* (up to 9.8% of Kraken2-classified reads), *Corynebacterium* (up to 56% of Kraken2-classified reads), *Oligella* (up to 3.7%) and *Porphyromonas* (up to 1.1%).

The microbial genera identified by both direct read classification approaches, namely Kraken2 and Metaphlan4, but not by metagenomic binning, can also be characterized by either high average abundance or the fact that most of them were present in at least one third of the analyzed samples. Genera *Pseudomonas* and *Mahnheimia* were the most abundant, share of the reads assigned to these genera reached 76 and 82%, respectively and they were detected in at least one third of the samples (Figure 3). *Cutibacterium* representatives, on the species level mainly determined as *С. acnes*, reached 12.9 and 21.1% of abundance as detected by Metaphlan4 and Kraken2, respectively. The genus *Moraxella*, represented by *Moraxella bovis* and *Moraxella ovis* species, was also present in a significant number of samples (up to 50% of the samples according to Kraken2 results), its numbers reached 29% of the total number of classified reads. Bacterial genera detected using both read classification algorithms, however, occurring in low numbers (less than 1 %) or detected in only one sample also included genera of opportunistic pathogens such as *Treponema, Acinetobacter, Mammaliicoccus, Acinetobacter, Micrococcus, Bibersteinia, Proteus* etc. (Figure 3).

The representation of bacteria detected using only one of the three algorithms in most cases did not exceed an average of 1% of the total number of classified reads. At the same time, a number of these species were also described as opportunistic pathogens (*Finegoldia*, *Malassezia*, etc.). Metagenomic bins obtained by *de novo* assembly included one non-cultivated genus of *Aerococcaceae* family, namely uncultured *Aerococcaceae* genus DSM-111022, which is included in the GTDB database (71), but is absent in the classical Kraken and Metaphlan databases, and as a result was not detected by direct classification of reads. However, all metagenomic genomes of this genus represented in GTDB were collected from raw milk samples,^1^ indicating the validity of this result.

### 3.3 *De novo* assembly results and phylogenetic analysis of high quality metagenomic bins

Due to the distant locations of the sampling sites and the observed diversity of strains, we conducted *de novo* assembly for each sample individually. However, due to inconsistent levels of contamination of metagenomic material with host genomic DNA, the quality and size of the assemblies varied considerably between samples (Table 4). Nonetheless, we selected the high-quality assemblies for metagenomic binning. As a result, we obtained 10 bins of quality, sufficient to obtain its taxonomic classification using GTDB-toolkit (Table 5). Four high-quality metagenomic bins (completeness >85%, contamination <5%) were generated through metagenomic binning and subsequently utilized for comprehensive phylogenetic analysis.

**Table 4:** Results of *de novo* assembly of metagenomic reads.

Assembly name	Millions of input reads	Thousands of contigs	Total length, Mbases	Max length, kbases
LSDV_88	0.73	38.07	12.39	112.64
LSDV_89	9.15	155.65	61.47	129.42
LSDV_90	1.57	110.21	35.75	5.69
SPV_92	2.52	5.65	1.73	6.37
SPV_93	1.37	14.15	4.43	96.54
SPV_94	2.17	4.26	1.34	41.96
SPV_95	2.95	16.77	9.38	62.62
SPV_96	6.86	2.39	0.57	2.65
SPV_97	0.31	5.1	1.4	0.94
SPV_98	5.28	17.94	5.71	2.9
SPV_99	1.44	13.98	3.94	2.48
SPV_100	4.77	144.93	57.03	134.05

**Table 5:** Obtained bacterial metagenomic bins.

Bin ID	Completeness	Contamination	Phylum	Family	Genus/Species
LSDV_89.metawrap.bin.2	99.57	2.01	Firmicutes	*Streptococcaceae*	*Streptococcus dysgalactiae*
LSDV_89.metawrap.bin.4	99.31	4.62	Firmicutes_A	*Peptoniphilaceae*	*Helcococcus ovis*
LSDV_89.concoct.bin.0	98.36	0.17	Bacteroidota	*Porphyromonadaceae*	*Porphyromonas_A* sp.
LSDV_89.concoct.bin.4	89	1.72	Bacteroidota	*Bacteroidaceae*	*Bacteroides* sp.
SPV_100.concoct.bin.18	75.26	20.8	Actinobacteriota	*Mycobacteriaceae*	*Corynebacterium* sp.
SPV_100.pyyamb.bin.3	47.33	8.95	Proteobacteria	*Burkholderiaceae*	*Oligella* sp.
SPV_95.concoct.bin.9	26.76	0.01	Fusobacteriota	*Fusobacteriaceae*	*Fusobacterium_C* sp.
SPV_95.pyyamb.bin.0	21.67	0.51	Firmicutes	*Staphylococcaceae*	*Staphylococcus* sp.
SPV_100.pyyamb.bin.2	19.76	0.65	Firmicutes	*Aerococcaceae*	*DSM-111022* sp.
LSDV_89.concoct.bin.5	19.66	0.02	Actinobacteriota	*Actinomycetaceae*	*Trueperella pyogenes*

Bin, classified as *Helcococcus ovis*, exhibiting 99.31% completeness and 4.62% contamination (Table 4), was used for phylogenetic analysis based on amino-acid sequences of 41 marker genes. The phylogenetic analysis confirmed the initial classification; however, it showed slight divergence from other *H. ovis* genomes isolated from the vagina of female cattle with metritis (72) (Supplementary Figure 3). Another high-quality MAG was classified as *Streptococcus dysgalactiae* also detected in high numbers by direct read classification approaches. Since there are a large number of *Streptococcus dysgalactiae* genomes in the NCBI database (185 genome assemblies in RefSeq as accessed on 01.09.2023), a multilocus sequence typing approach was used to obtain a high-resolution phylogeny of this metagenomic bin. This metagenomic bin clustered with the genomes of *S.dysgalactiae* subspecies *dysgalactiae* (73) obtained in two large-scale studies aimed at investigating the phylogeny and virulence factors of S.dysgalactiae strains associated with bovine mastitis. In these two studies, one in the United States (74) and the other in China (unpublished),^2^ *S. dysgalactiae* strains were isolated from the milk of cows with mastitis. All other genomic sequences in this cluster except those assembled from the type material were also associated with bovine mastitis (Supplementary Figure 4).

Bin classified by GTDB-toolkit as *Porphyromonas_A*, a new genus, recently reclassified from classical *Porphyromonas* genus by genome-based taxonomy approach (71), was placed through *de novo* phylogenetic reconstruction in close proximity to the valid species *Porphyromonas bennonis*, isolated from human clinical specimens, such as cysts and abscesses (75). Other metagenome-derived GTDB reference species, closely related to this metagenomic bin, were assembled from mammal microbiome specimens (Supplementary Figure 5). Analysis of the fourth high-quality metagenomic bin belonging to *Bacteroides* genus (89% of completeness and 1.72% of contamination) through comparison of average nucleotide identity with other members of the genus (Supplementary Figure 6) indicated its closest relation with the *Bacteroides heparynolyticus* species linked to human periodontal inflammation (76). However, it must be pointed out that ANI value between LSDV_89.concoct.bin.4 and *B. heparynolyticus* genome was only 92.26%, which is substantially lower than the intraspecies value commonly acknowledged (71, 77). Nonetheless, the quality of this MAG is not sufficient to propose a new *Bacteroides* species.

### 3.4 Analysis of antibiotic resistance genes in obtained metagenomic MAGs

Search for antibiotic resistance genes in identified MAGs performed with *staramr* package (53) showed only one tet(W) tetracycline resistance cluster in metagenomic bin, corresponding to unidentified *Oligella* (Table 5).

## 4 Discussion

### 4.1 Identification of co-infections using metagenomic approach

Metagenomic profiling of co-infections has recently been introduced into clinical practice as a result of the decreased cost of Next-Generation Sequencing (NGS) technologies in last decade (78). NGS analysis provide unprecedented data at the pan-genomic level, giving an opportunity to analyze all potential pathogens in one dataset (79, 80). The key benefit of shotgun metagenomic analysis, in contrast to analysis of microbial community composition using marker genes (16S rRNA), is that it allows for the simultaneous detection of all types of pathogens, including viruses, bacteria, and eukaryotic microorganisms (81). At the same time, the high cost of shotgun sequencing does not make it a universal method for screening coinfections. In addition to the fact that analysis of complete metagenomes requires obtaining a large number of reads, the DNA of microbial/viral communities is always contaminated with host DNA, with contamination levels often reaching 95–99% (82). In this context, it is crucial to choose data analysis methods that balance two key requirements: high sensitivity for detecting low-represented microorganisms and high specificity to minimize false positives.

In our study three different methods were employed examine the composition of microbial communities from skin scabs/lesions of SPV-infected and LSDV-infected animals. Classification of reads using Kraken2 predicted the presence of the largest number of taxonomic groups in the community—172 microbial genera were identified (Figure 3). This result aligns with previous benchmark studies (83, 84) that have found Kraken 2 to be the most sensitive classification algorithm used in metagenomic research. However, even during the early stages of analysis, it was observed that this algorithm can produce false positive outcomes. Thus, employing the standard extended Kraken2 database, the presence of highly virulent pathogen *Clostridium botulinum* was predicted in practically all samples reaching 80% abundance. However, the analysis of *de novo* assemblies and marker genes failed to confirm the pathogen’s presence. Further examination of reads attributed to *C.botulinum* demonstrated that they originated from mammalian genomes (data not shown). Including the host genomes in the custom Kraken database, as outlined in the Methods section, confirmed that *C.botulinum* presence was a false positive. Another false positive result generated by Kraken2 involves the detection of a significant number of reads linked to the eukaryotic pathogen *Babesia bigemina* (Figure 2). However, this outcome also lacked support from other methods. After additional analysis, it was discovered that none of the *Babesia*-related reads match any chromosomes or long scaffolds. Instead, they arise from two short contigs that appear to have resulted from contamination of the *B. bigemina* genome, deposited in NCBI. It is worth noting that this observation is not influenced by algorithm bias. Instead, it is due to insufficient inspection and purging of contamination even from the RefSeq database.

Apparently, examining the existence of clade-specific marker genes through direct mapping of reads to their nucleotide sequences can counterbalance the biases that come with the misassignment of k-mers to a specific genome and with the pollution of publicly available genomes in databases. In fact, the analysis employing Metaphlan4, which enabled the detection of 54 genera in a complete dataset, indicated that the majority of microorganisms were either significantly abundant or appeared in a large number of samples. Due to its comparatively lower computational requirements, it can be reasonably assumed that Metaphlan4 is the preferred direct-read classification method for clinical applications when compared to Kraken2. It is worth mentioning that Metaphlan generates Taxonomic abundance by mapping reads to single-copy marker genes. The number of mapped reads corresponds to the number of genome copies in the sample. On the other hand, Kraken2/Bracken outcomes are not normalized by genome size, and therefore show sequence abundance, representing the amount of DNA from a particular organism in the sample (85). The Kraken profiling outcomes may largely distort the actual representation pertaining to the ratio of microorganisms possessing very different genome sizes, e.g., eukaryotic and prokaryotic microorganisms, or bacteria and viruses. In scenarios involving complex microbial communities consisting of diverse viruses, pro- and eukaryotes, it is almost impossible to reliably convert sequence abundance values into taxonomic abundance (86). When studying the dynamics of microbial communities, it is crucial to accurately track the relationships between various (micro)organisms. Therefore, the recommended method is to utilize algorithms that rely on the mapping of reads to marker genes. The primary aim of this study is to characterize the microbial communities of scabs, focusing on the dominant microbes. In this vein, it is crucial to eliminate false positives and negatives, and therefore the use of two alternative algorithms to classify reads and compare results provides the most reliable approach.

However, it must be noted that a significant limitation of Metaphlan is its reliance on the human microbiome to form its database (34). Although it may have widespread applicability among mammals, its effectiveness with other organisms is uncertain and may yield false negative results (56).

*De novo* assembly, binning, and acquiring high-quality metagenome-assembled genomes (MAGs) are undoubtedly the gold standard of metagenomic analysis. Nonetheless, generating high-quality MAGs necessitates substantial sequencing depth as well as having multiple samples from the same environment for differential coverage binning. Unfortunately, in the case of metagenomic analysis of clinical samples, this is usually impossible as samples from different individuals and geographical locations cannot be combined due to strain-specific differences. Even minor dissimilarities in the genome sequences of one species can result in inadequate assembly quality, and consequently, uncertain outcomes. In the future, progress in long-read technologies, such as Oxford Nanopore, could potentially resolve this issue. Using hybrid assembly, longer contigs and high-quality MAGs can be obtained without the need for binning through differential coverage (87). However, in this work *de novo* assembly did not bring the expected results. Firstly, due to elevated levels of host DNA contamination and therefore insufficient number of target reads, the assembly turned out to be fragmented and the length of contigs was insufficient for reliable taxonomic classification or binning. Secondly, differential binning was not feasible as only a single sample per assembly was used. Nonetheless, we acquired 10 metagenomic bins and managed to establish their taxonomic position through genomic taxonomy. Out of these, four were high-quality bins, and we accurately established their taxonomic position and relatedness to opportunistic strains using classical phylogeny methods such as phylogeny based on marker genes, MLST, and ANI analysis.

The findings of this study reveal that although Kraken2 boasts high sensitivity, the prevalence of false positives raises concerns about its feasibility in practical settings. However, it must be noted that while Metaphlan4 boasts high specificity, it may struggle to detect low-represented microorganisms. Furthermore, the specificity of the marker gene database used by Metaphlan in relation to the human microbiome presents a challenge in its application to veterinary medicine. In turn, *de novo* assembly is the most reliable and specific method, however it necessitates a significant amount of sequencing data. To a certain extent, this issue can be resolved by removal of host DNA before sequencing, either through differential cell lysis or through the removal of mammalian DNA by methylcytosine-binding protein (88). At the same time, one clear advantage of *de novo* assembly is the potential to accurately predict the resistance of opportunistic strains to antibiotics, enabling the appropriate choice of antibiotic therapy.

While metagenomic approaches are actively being developed for veterinary research, practical guidelines for the standardization of bioinformatics analysis are currently lacking (89). Thus, researchers must decide on the sensitivity and specificity of the algorithms used, depending on the quality and quantity of primary data in the context of the research objectives. An equally significant factor when choosing algorithms and databases is the accessibility of computing power, particularly the amount of RAM. According to the literature, optimal sensitivity and specificity can be attained by employing nucleotide databases that encompass all living kingdoms, which demands a vast amount of memory (56). Unfortunately, at present, a universal solution for bioinformatics analysis of clinical metagenomic samples cannot be provided. However, based upon the data and results of analysis presented here, we suggest the decision-making scheme illustrated in Supplementary Figure 6.

### 4.2 Taxonomic composition of scab’s microbial communities

In this study we characterized the microbial community composition of scabs developed in capripoxvirus-related infections and evaluated the presence of opportunistic pathogens. It should be noted that although various studies have investigated the microbiome and its possible contribution to coinfection development for various cattle diseases (90, 91), most have been conducted in controlled experimental settings and never with capripoxvirus infections. Therefore, this study, which analyses the microbiome of skin lesions in viral infections of farm animals using real clinical specimens, is one of the first of its kind performed by shotgun sequencing (92). Unfortunately, due to strict selection criteria for sample conditions and logistical challenges in obtaining clinical samples from remote regions, the number of samples involved in this study was limited. Therefore, the authors acknowledge that this study is a pilot, providing initial insights into the microbial diversity of skin lesions. However, it does not allow for a quantitative assessment of microbiome changes. Moreover, comparing the data obtained with other studies on the microbiome of cutaneous lesions in farm animals with capripoxvirus infections is not feasible due to the lack of information on this matter. Therefore, although the data obtained in this study is highly valuable, it is necessary to carry out more extensive studies, including longitudinal studies, to accurately analyze the diversity and dynamics of the microbial communities of skin scabs and their role in the pathogenesis of capripoxvirus infections.

The investigation of microbial community alpha- and beta-diversity in scabs/lesions revealed that, despite the established differences between SPV and LSDV (93), as well as the skin microbiome of their respective hosts (94, 95), no substantial differences were identified between LSDV or SPV-related lesions. Nevertheless, the diversity index values were inferior to those reported in literature for healthy skin (59). This suggests that the composition of specific microbial communities of scabs formed during capripoxvirus infection is predominantly influenced by one or two prevailing opportunistic microorganisms. It should be noted, that since several studies have reported an association of alpha diversity of microbial communities of disease-affected organs with immune response and disease severity (96, 97), analyzing alpha diversity on a more expanded dataset compared to control samples of healthy skin sites may provide additional insights into the mechanisms of severe symptom development.

As demonstrated by direct read classification, viral DNA was the predominant source of the sequencing reads that were not related to host DNA, reaching for 92% of those (Figure 2). Moreover, considering the biases in Kraken2-based sequence abundance associated with the small genome size of capripoxviruses, we can assume that the true number of copies of viral genomes is absolutely prevalent in the community. In a single instance, co-infection of SPV with orf virus, was identified. Orf virus is commonly found as a co-infection in several viral pathologies (98, 99), although its exact impact on the development of symptoms remains uncertain. In turn, there was a great deal of variation in the composition of bacterial communities that develop in samples from sheep and cows. Bioinformatic analysis of prokaryotic part of the scab’s communities indicated that pattern of prevailing taxa is highly flexible. This is likely due to variability of dominating commensal bacteria in specific skin area. Nonetheless, analysis of core community members identified few genera detected in substantial amounts in at least half of the examined samples (Table 3).

The most prevalent coinfection identified was *Fusobacterium necrophorum*, which is known to cause several severe conditions in livestock, including foot abscess, bovine liver abscess, and calf diphtheria (100). Another prevailing pathogen was *Streptococcus dysgalactiae*, which was also confirmed through *de novo* metagenomic assembly and binning, was found in nearly all samples. This bacterium has the potential to cause opportunistic infections in both animals and humans in various body sites (63, 101). A thorough investigation of the microorganism’s phylogenetic position using MLST demonstrated that the identified strain belongs to the same cluster as the genomes of *S.dysgalactiae* obtained from cows’ milk with mastitis (74). This fact, along with abundant evidence linking *S. dysgalactiae* to mastitis (74, 102), indicates that the mastitis symptoms described in certain capripoxvirus infections (103) are caused by this opportunistic pathogen. The same can be said for another bacterium, *Helcococcus ovis*, associated with metritis in cattle and sheep (66, 104). There is evidence to suggest that metritis frequently develops as a complication of LSDV infection (105, 106). Thus, it is probable that, as with *S. dysgalactiae*, the virus is not the direct causative agent of this symptom, but rather the bacterial coinfection.

*Trueperella pyogenes,* which was present in relatively small numbers but detected in more than half of the samples, is also a common commensal causing a variety of purulent infections in livestock (107). The correlation between the presence of this pathogen and *F.necrophorum* during the development of footrot disease in sheep, described by Usie et al. (91), suggests the development of a complex co-infectious process in scabs.

Other commonly occurring microorganisms found in a considerable number of samples belong to the genera Bacteroides and Porphyromonas_A, as per the GTDB taxonomy (71). However, phylogenetic analysis of their MAG’s suggests that they may fall under new, unreported species of said genera. Therefore, their potential contribution to the onset of symptoms resulting from SPV/LSDV infection is yet to be determined.

Taken together, the results of this study suggest that the scabs develop an active coinfection process due to opportunistic pathogens. The presence of bacterial coinfection is likely to be responsible for severe secondary symptoms associated with LSDV/SPV infection. This is in agreement, in part, with the research conducted by Usie et al. who utilized shotgun metagenomics of skin lesions to reveal the multibacterial nature of sheep footrot (91). It is noteworthy that in the mentioned study the metagenome was analyzed under experimental conditions, enabling the evaluation of process dynamics. The present research, however, involved direct analysis of clinical samples, leading to discussions on the practicability of the shotgun-metagenomic approach in the clinical setting. It is noteworthy that the search for antibiotic resistance genes in the acquired MAGs suggests that these strains should be susceptible to antibiotic therapy. This finding supports the rationale for implementing supplementary antibiotic treatment in cases of capripoxvirus infection.

At the same time, the author should emphasize the limitations of this study due to its design and subject matter. The use of clinical samples collected in real field conditions allows for a maximization of the approximation of experimental conditions to the practical conditions in which veterinarians work. However, due to the absence of comparison groups (except for grouping samples according to infectious agent) and the lack of continuous sampling during the progression of the disease, it is impossible to conduct functional analysis of the metagenome and dynamic analysis of microbiota changes. Nevertheless, presented findings provide a promising foundation for further research into the pathogenesis of capripoxvirus-related infections.

## Data availability statement

The raw read files and high-quality MAGs were deposited at DBJ/EMBL/GenBank under the Bioproject PRJNA1023210.

## Ethics statement

Ethical approval was not required for the study involving animals in accordance with the local legislation and institutional requirements because this study was based solely on skin scab samples submitted to FGBI ARRIAH for confirmation of capripoxvirus infection, thus no animal experiments were performed as part of this work.

## Author contributions

FS: Data curation, Investigation, Software, Visualization, Writing – original draft, Writing – review & editing. AM: Investigation, Methodology, Resources, Writing – original draft, Writing – review & editing. AOK: Investigation, Methodology, Resources, Writing – review & editing. OB: Investigation, Methodology, Resources, Writing – review & editing. LP: Project administration, Resources, Writing – review & editing. IC: Conceptualization, Funding acquisition, Writing – review & editing. UZ: Writing – review & editing, Data curation, Investigation, Software. ADK: Data curation, Software, Writing – review & editing. ASK: Investigation, Methodology, Writing – review & editing. EG: Writing – review & editing, Project administration, Resources. AP: Writing – review & editing, Investigation, Methodology. AAK: Writing – review & editing, Data curation, Software. MP: Writing – review & editing, Funding acquisition, Project administration. ZN: Writing – review & editing. AS: Conceptualization, Supervision, Writing – original draft, Writing – review & editing, Funding acquisition, Investigation, Project administration. ST: Conceptualization, Data curation, Methodology, Software, Supervision, Visualization, Writing – original draft, Writing – review & editing.

## Footnotes

^1^https://gtdb.ecogenomic.org/genome?gid=GCA_015863195.1

^2^https://www.ncbi.nlm.nih.gov/bioproject/PRJNA753266
