# Supplementary material for: Metagenomic profiling of viral and microbial communities from the pox lesions of lumpy skin disease virus and sheeppox virus-infected hosts
Source: Front Vet Sci. 2024 Feb 14;11:1321202. doi: 10.3389/fvets.2024.1321202 (PMC10899707; doi:10.3389/fvets.2024.1321202)
Supplement: Supplementary file 1 [file Data_Sheet_1.zip › Tables S1,2,4&5 & Figures S1-6.DOCX]

Supplementary Material

# Supplementary Tables

**1.1 Supplementary Table 1.** Percentage of filtered reads classified by Kraken2 and Metaphlan4

| Sample ID | % of reads classified by Kraken2 (in total) | % of reads classified by Kraken2 as non-host-related | % of reads classified by Metaphlan4 |
| --- | --- | --- | --- |
| LSDV_88 | 28.05 | 12.99 | 0.03 |
| LSDV_89 | 36.73 | 19.55 | 6.45 |
| LSDV_90 | 14.7 | 9.35 | 0.18 |
| SPV_92 | 33.51 | 4.8 | 0.35 |
| SPV_93 | 67.2 | 55.71 | 27.77 |
| SPV_94 | 27.97 | 7.64 | 0.38 |
| SPV_95 | 38.4 | 19.62 | 9.8 |
| SPV_96 | 28.98 | 5.29 | 0.48 |
| SPV_97 | 16.25 | 8.27 | 1.54 |
| SPV_98 | 64.98 | 50.47 | 73.84 |
| SPV_99 | 29.01 | 8.03 | 1.01 |
| SPV_100 | 28.32 | 10.6 | 7.69 |

**Table 2.** Alpha diversity metrics

| SAMPLE ID | number of species detected by Kraken2 | number of species detected by Metaphlan4 | shannon index, based on Kraken2 results | shannon index based on Metaphlan4 results | number of reads used for classification |
| --- | --- | --- | --- | --- | --- |
| LSDV_88 | 5 | 1 | 1.261 | 0 | 0.78 |
| LSDV_89 | 78 | 17 | 1.878 | 1.541 | 9.25 |
| LSDV_90 | 9 | 2 | 1.449 | 0.368 | 1.67 |
| SPV_92 | 17 | 5 | 1.432 | 1.168 | 13.49 |
| SPV_93 | 87 | 30 | 2.503 | 2.442 | 5.53 |
| SPV_94 | 13 | 5 | 0.87 | 0.655 | 11.8 |
| SPV_95 | 67 | 24 | 1.834 | 1.739 | 13.65 |
| SPV_96 | 28 | 16 | 2.571 | 2.212 | 20 |
| SPV_97 | 13 | 2 | 1.825 | 0.385 | 6.38 |
| SPV_98 | 268 | 23 | 3.588 | 1.614 | 10.02 |
| SPV_99 | 20 | 8 | 2.085 | 1.514 | 16.34 |
| SPV_100 | 130 | 56 | 3.422 | 2.846 | 7.08 |

**Table 3.** Results of Metaphlan4 classification on species level

Table is presented as separate Excel spreadsheet, SupTable3.metaphlan.xlsx

**Table 4.** Results of permutational analysis of variance using Bray distance matrices, performed with *adonis2* function of *vegan* package.

| **Kraken2** | Call: adonis(formula = k2.bug_mat ~ virus, data = metadata) | | | | | | |
| --- | --- | --- | --- | --- | --- | --- | --- |
|  | Permutation: free; Number of permutations: 999 | | | | | | |
|  |  | **Df** | **SumsOfSqs** | **MeanSqs** | **F.Model** | **R^2^** | **Pr(>F)** |
|  | **virus** | 1 | 0.5259 | 0.52588 | 1.289 | 0.11418 | 0.106 |
|  | **Residuals** | 10 | 4.0799 | 0.40799 |  | 0.88582 |  |
|  | **Total** | 11 | 4.6058 |  |  | 1.00000 |  |
| **Metaphlan4** | Call: adonis(formula = bug_mat ~ virus, data = m4.metadata) | | | | | | |
|  | Permutation: free; Number of permutations: 999 | | | | | | |
|  |  | **Df** | **SumsOfSqs** | **MeanSqs** | **F.Model** | **R^2^** | **Pr(>F)** |
|  | **virus** | 1 | 0.6267 | 0.62671 | 1.441 | 0.12595 | 0.048 |
|  | **Residuals** | 10 | 4.3492 | 0.43492 |  | 0.87405 |  |
|  | **Total** | 11 | 4.9759 |  |  | 1.00000 |  |

**Table 5. Results of analysis of Kraken2-сlassified reads assigned to *Babesia bigemina*.**

| **Sample** | **Babesia reads by Kraken** | **Alignment rate** | **Babesia chromosomes** | **scaffold Bbigscaff_63091** | **scaffold Bbigscaff_63120** | **scaffoldBbigscaff_63221** | **scaffold Bbigscaff_8321** | **scaffold Bbigscaff_8367** | **Other Babesia scaffolds** |
| --- | --- | --- | --- | --- | --- | --- | --- | --- | --- |
| LSDN_88 | 898 | 99.72% | 0 | 507 | 90 | 23 | 179 | 96 | 0 |
| LSDN_89 | 15169 | 99.89% | 0 | 9994 | 2156 | 393 | 2273 | 336 | 0 |
| LSDN_90 | 2087 | 99.88% | 0 | 695 | 572 | 48 | 623 | 146 | 0 |
| SPV_92 | 1074 | 99.72% | 0 | 12 | 421 | 22 | 507 | 108 | 0 |
| SPV_93 | 116 | 100.00% | 0 | 4 | 32 | 0 | 56 | 24 | 0 |
| SPV_94 | 1580 | 99.91% | 0 | 8 | 615 | 58 | 686 | 211 | 0 |
| SPV_95 | 892 | 99.89% | 0 | 10 | 330 | 16 | 394 | 141 | 0 |
| SPV_96 | 6188 | 99.88% | 0 | 12 | 2964 | 134 | 2286 | 784 | 0 |
| SPV_97 | 109 | 99.54% | 0 | 20 | 35 | 4 | 37 | 12 | 0 |
| SPV_98 | 92 | 98.91% | 0 | 22 | 28 | 0 | 14 | 26 | 0 |
| SPV_99 | 1021 | 99.90% | 0 | 14 | 303 | 18 | 507 | 177 | 0 |
| SPV_100 | 730 | 100.00% | 0 | 8 | 322 | 6 | 356 | 38 | 0 |

# Supplementary Figures


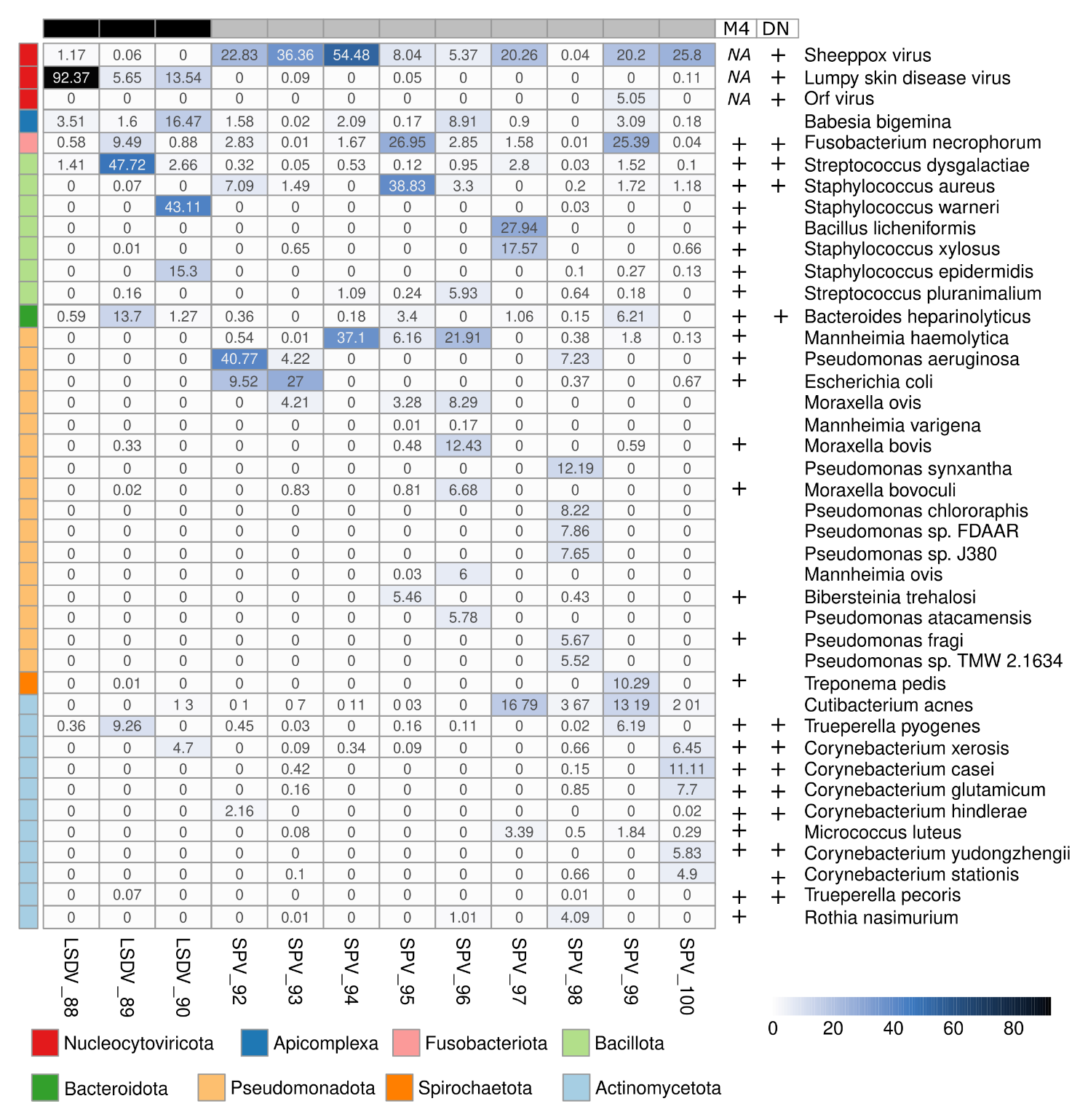


**Supplementary Figure 1.** Heatmap of the results of species-level Kraken2 classification.


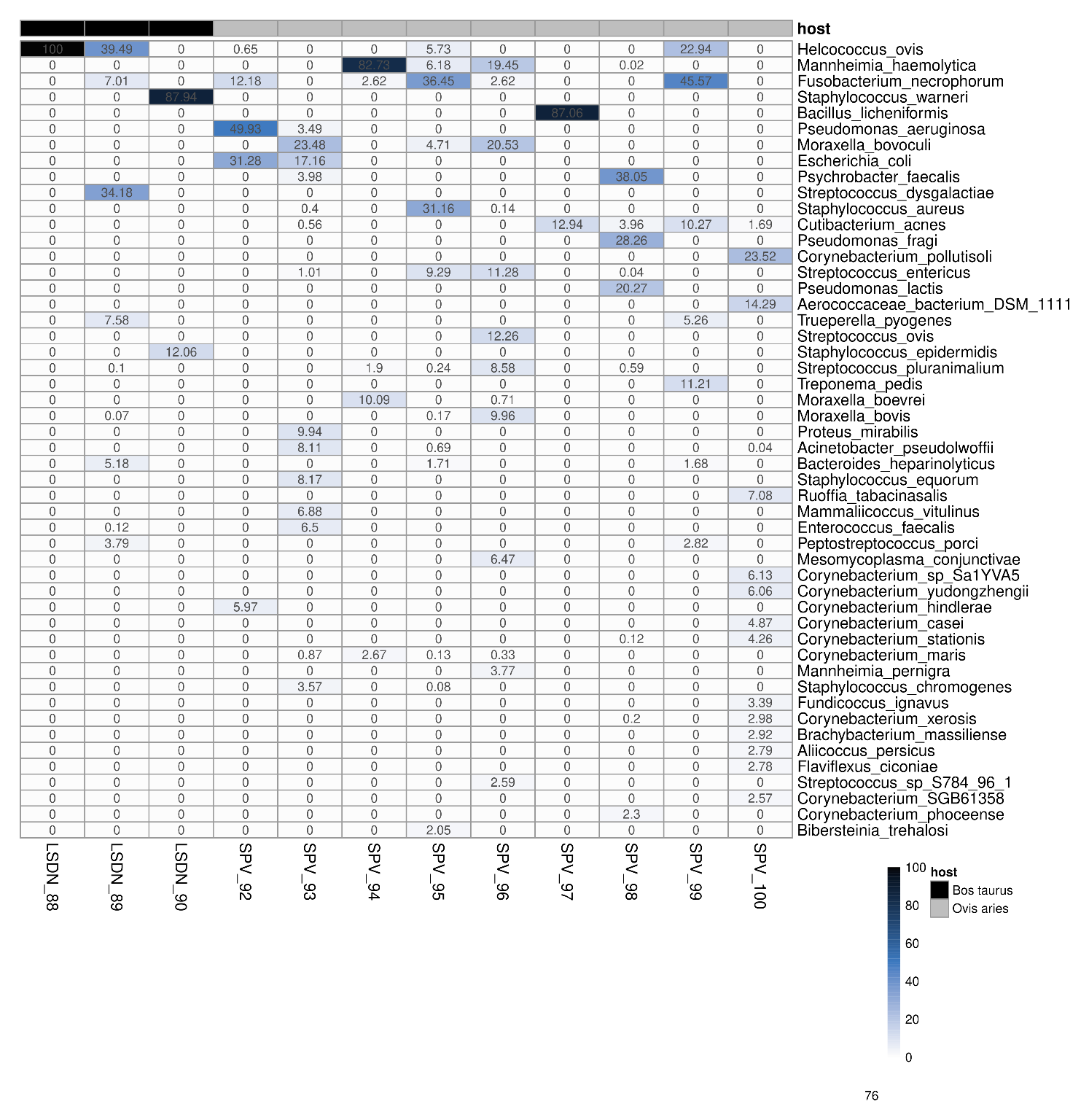


**Supplementary Figure 2.** Heatmap of the results of species-level Metaphlan4 classification.


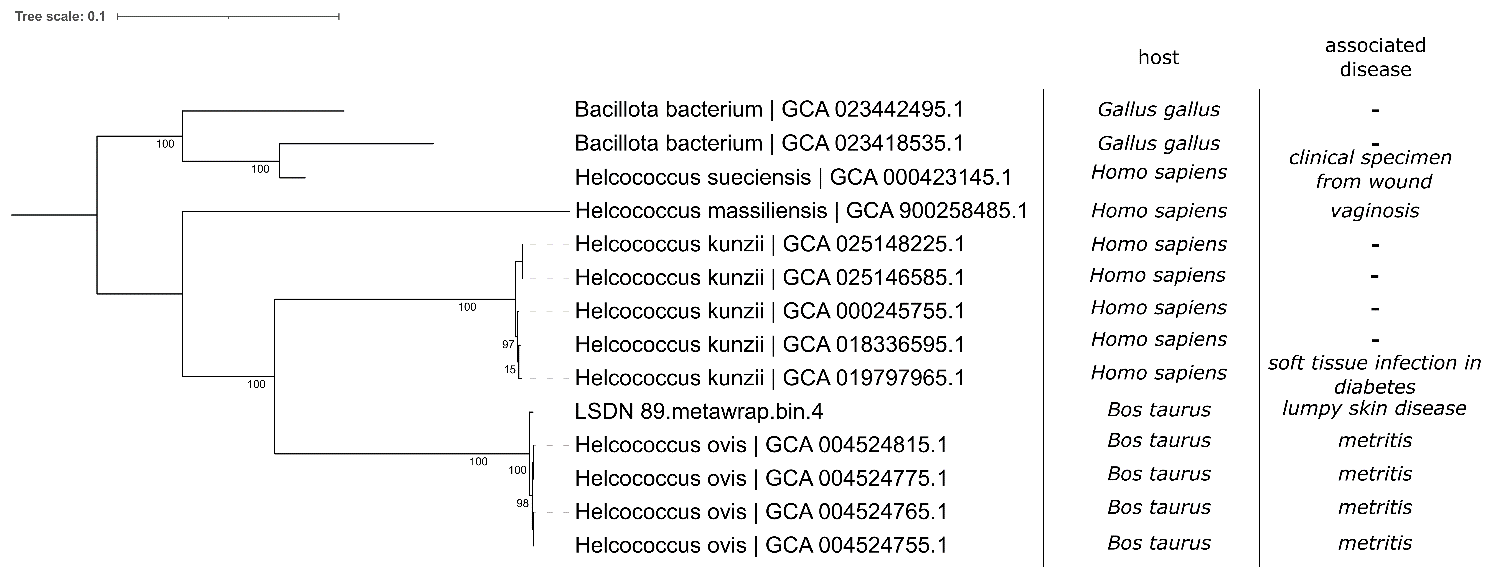


**Supplementary Figure 2.** Phylogenetic tree based on 41 single copy marker gene amino acid sequences of representatives of the *Helcococcus* genus (according to GTDB, accessed on 1st September 2023). The host and associated disease are shown on the right panel. Bootstrap support is indicated as percentage.


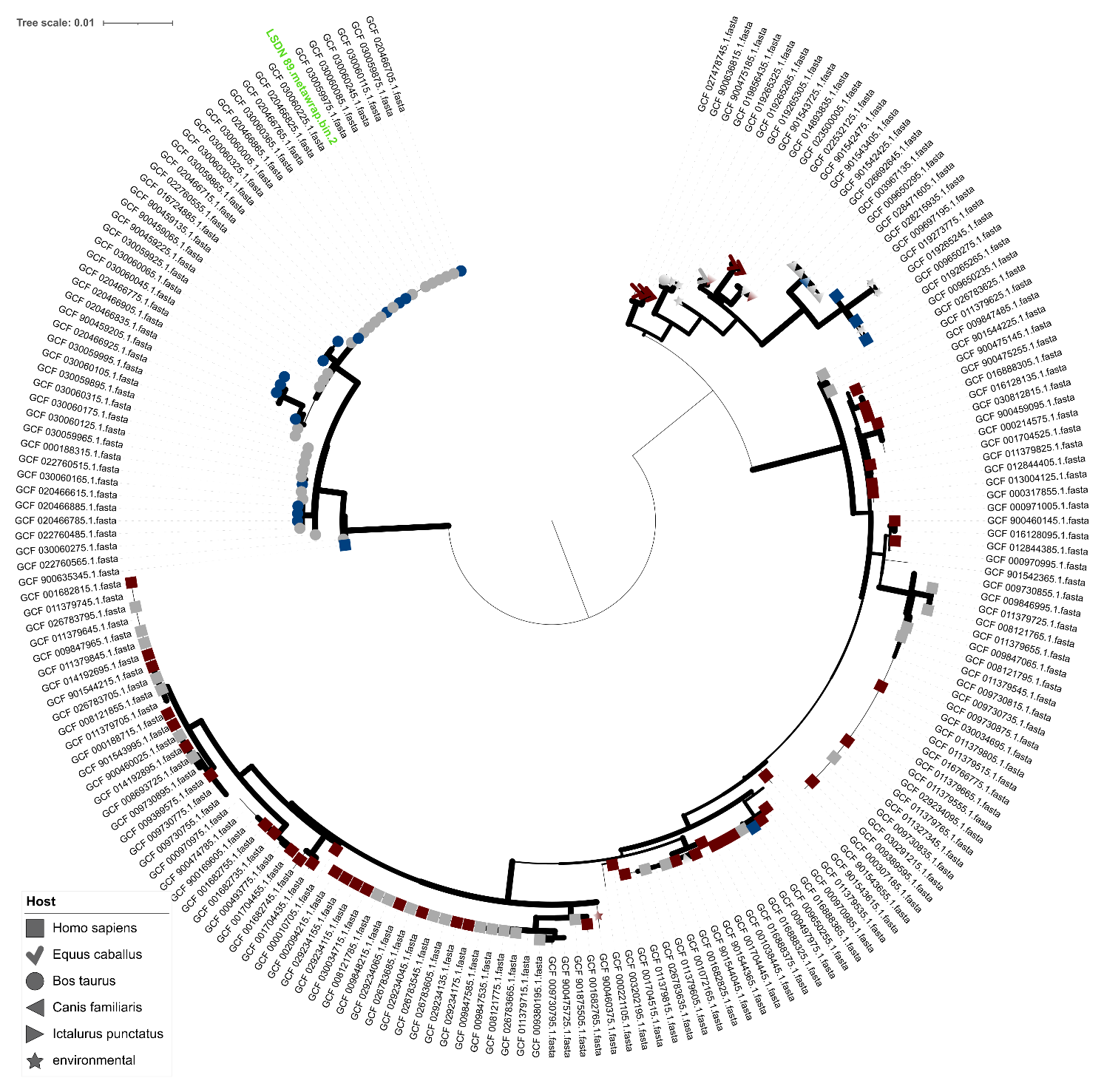


**Supplementary Figure 3.** Phylogenetic tree based on nucleotide sequences of multi-locus sequence typing scheme of Streptococcus dysgalactiae (according to https://pubmlst.org/, accessed on 27th August 2023). Bootstrap support is mapped as branch width.


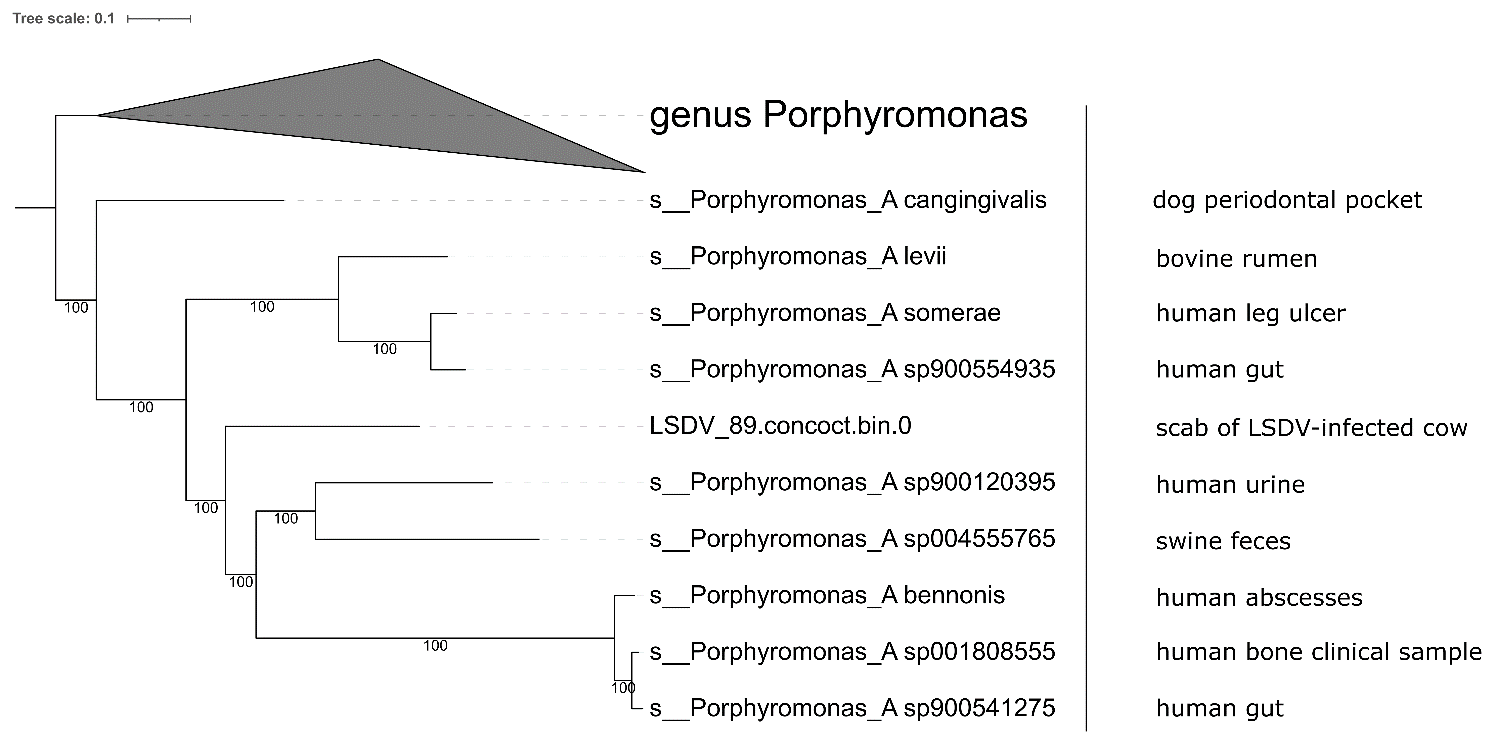


**Supplementary Figure 4.** Maximum likelihood phylogenetic tree based on 120 single copy marker gene amino acid sequences of representatives of the *Porphyromonas_A* genus (according to GTDB, accessed on 1st September 2023). Tree was reconstructed using GTDB-Tk *de novo* workflow using *Porphyromonas* representatives as the outgroup. Sample isolation sources are shown on the right panel. Bootstrap support is indicated as percentage.


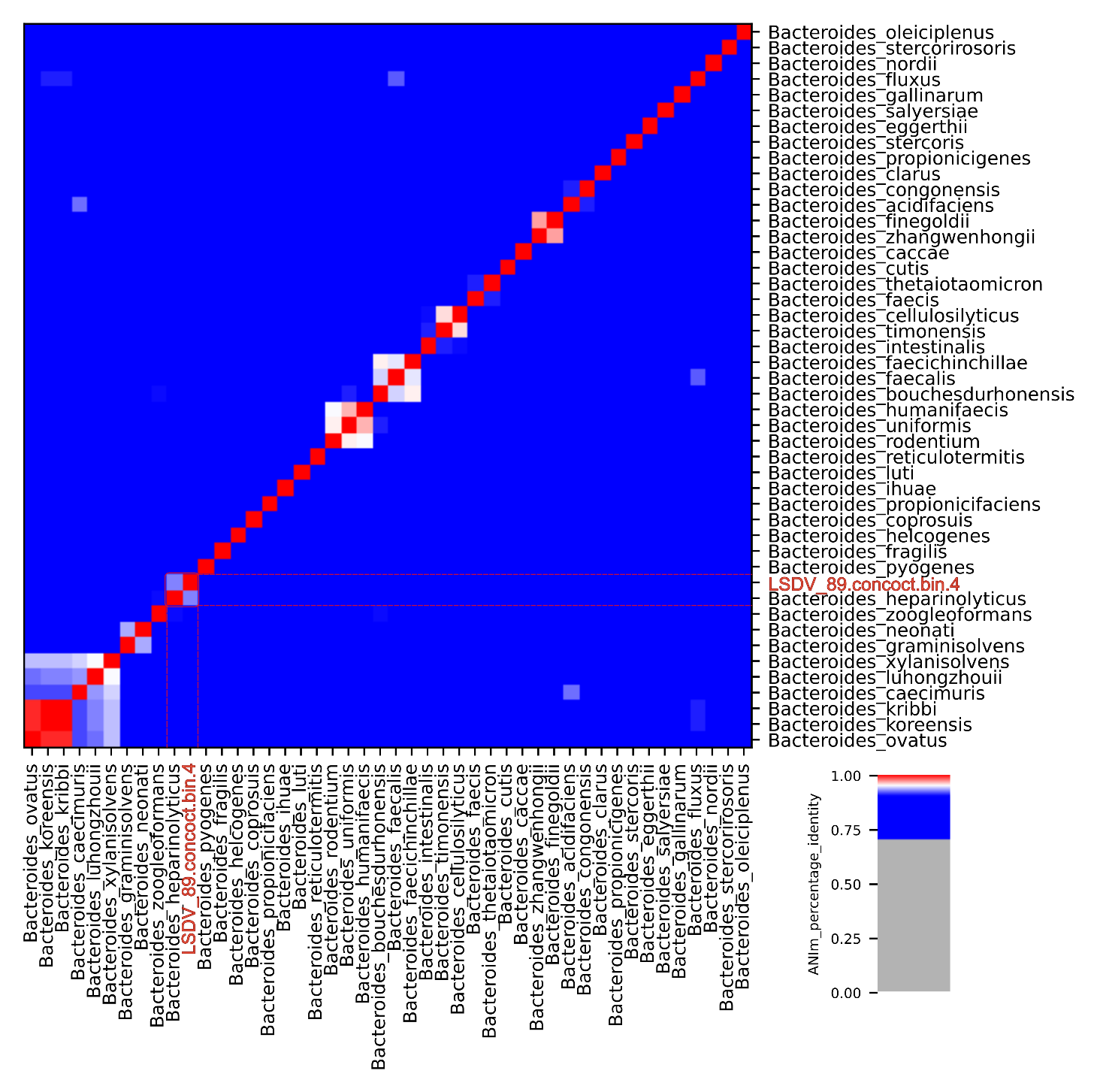


**Supplementary Figure 5.** Heatmap of average nucleotide identity of GTDB representative species of *Bacteroides* genus. Heatmap was drown with FastANI package.


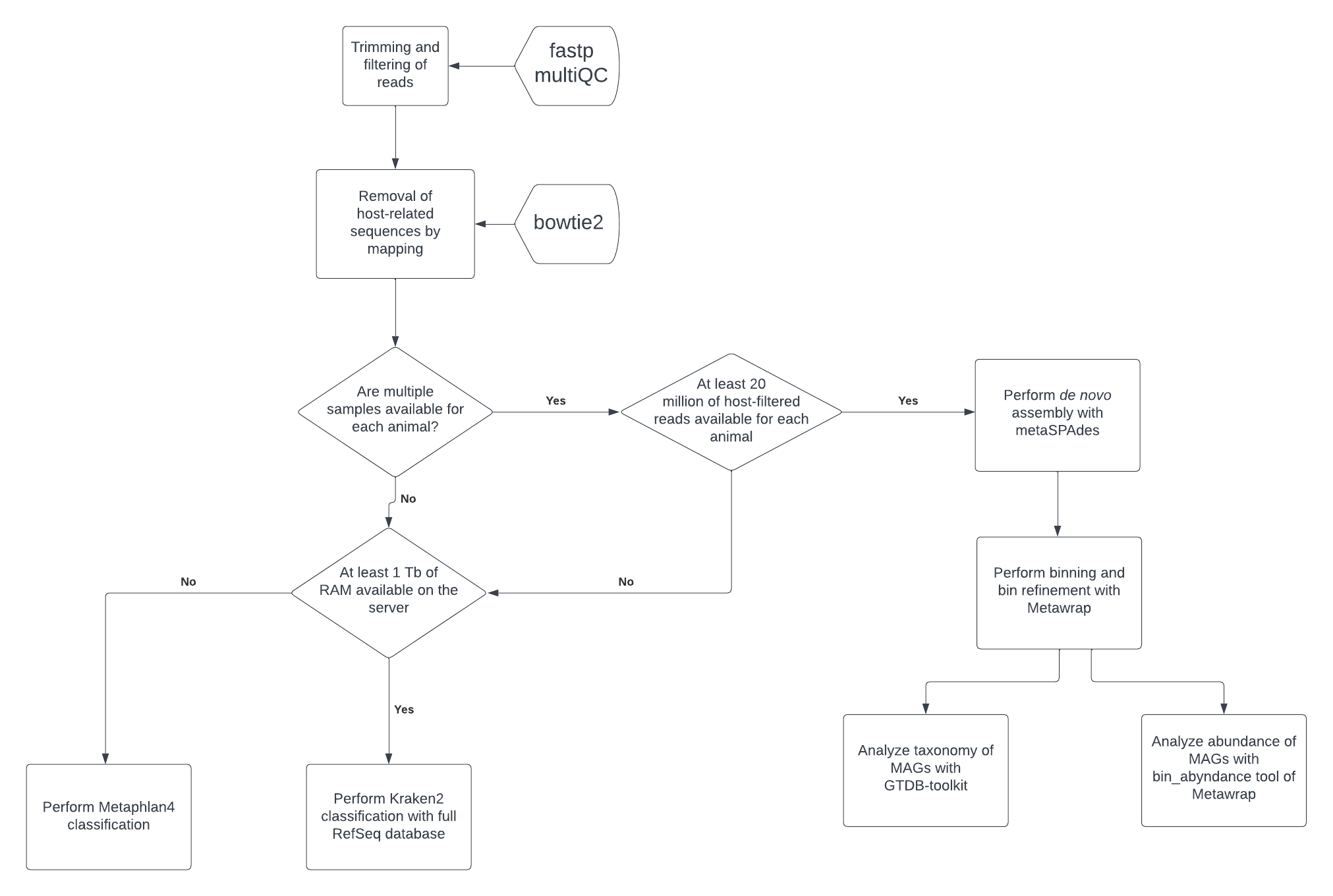


**Figure 6.** Decision chart for the bioinfomatic analysis of metagenomic data.
